# The ebbs and flows of empathy: a qualitative study of surgical trainees in the UK

**DOI:** 10.1186/s12909-024-05105-x

**Published:** 2024-02-09

**Authors:** Pranathi Yannamani, Nicola Kay Gale

**Affiliations:** 1 University of Birmingham Medical School, Birmingham, UK; 2 Health Policy and Sociology, Health Services Management Centre, School of Social Policy, University of Birmingham, Birmingham, UK

**Keywords:** Empathy, Surgical training, Interprofessional empathy, COVID-19

## Abstract

**Background:**

Empathy is widely recognised as an important element of medical practice contributing to patient outcomes and satisfaction. It is also an important element of collaborative work in a healthcare team. However, there is evidence to suggest that empathy towards patients declines over time, particularly in surgical specialities. There is little qualitative research on this decline in surgical trainees, particularly in the UK. Therefore, the aim of this study was to explore how trainee surgeons experience empathy over the course of their career, both towards patients and colleagues and how they perceive it in others.

**Methods:**

10 semi-structured interviews were carried out with surgical trainees of different grades and specialties in January and February 2022. Framework analysis was used to interpret the data.

**Results:**

Participants experienced an evolution in empathy over their career as their personal and professional experience was added to. They drew a distinction between desensitisation and actual decline in empathy and identified more with experiencing the former in their careers. Participants also felt interprofessional relationships require empathy, and this could be improved upon. Finally, they highlighted specific impacts of the COVID-19 pandemic upon their training, including reduced theatre time.

**Conclusions:**

Participants felt training could be improved in regard to accessing training opportunities and relationships with colleagues, although many felt empathy between colleagues is better than it has been in the past. This project highlighted areas for future research, such as with surgeons in later stages of their careers, or mixed-methods projects.

## Background

### Empathy in medical practice- for patients and colleagues

Empathy is an important factor in the way doctors practise medicine, as increased physician empathy has been shown to improve patient outcomes and satisfaction [1]. Physician empathy also reduces patient anxiety and increases their understanding and ability to cope with their condition [2]. In fact, regardless of the therapeutic context, empathy has been shown to foster communication between the healthcare professional and the patient, empower the patient and help resolve the patient’s problems [3]. The importance of empathy in medical education is recognised as it is a required component of medical training in the United Kingdom and the United States [4, 5].

While there is not one single definition of clinical empathy due to its ambiguity as a concept, a concise summary is psychologist Carl Rogers’ definition: “to sense the client’s private world as if it were your own, but without losing the ‘as if’ quality” [6].

In addition to empathy towards patients, interprofessional empathy is being increasingly recognised as important in collaborative patient care, but this link is less discussed in the literature [7]. Furthermore, studies have suggested that interprofessional relationships in healthcare are often characterised by hostility and conflict rather than cooperation [8–11]. Empathy towards colleagues is a little-researched area, despite teamwork being one of the foundational principles upon which healthcare works and empathy being a key component of teamwork [12].

### Empathy decline in surgeons

Much research has been conducted that shows a decline in empathy in medical students, beginning in their first year of clinical placement and continuing through medical training [13–17]. This decline in empathy is also associated with an increase in cynicism and emotional detachment [18].

The reasons for the empathy decline are unclear but influencing factors include discrimination or mistreatment by superiors, social support problems and a high workload [17]. These are examples of the ‘hidden curriculum’, influences that exist outside the official medical school curriculum and teaching but are nevertheless important to learn. As Hafferty describes them, “commonly held “understandings”, customs, rituals, and taken-for-granted aspects of what goes on in the life-space we call medical education” [19].

Students favouring technology-oriented specialties, or “patient-remote” specialties (e.g., surgery, radiology) had lower empathy scores than those favouring specialties with more patient interaction [17, 20]. The classification of certain specialties as ‘patient-remote’ in this literature is subjective, as there is arguably a significant amount of patient contact in surgical specialties. There is also research suggesting the empathy decline is more severe in surgery than in medicine, highlighting a potential need for empathy training [21]. Furthermore, surgeon empathy, as rated by patients, is the strongest motivator of patient satisfaction [22]. However, a 2008 study of orthopaedic surgeons’ consultations showed that patients only raised around half of their concerns with their surgeons, rarely raising social issues [23]. This could be due to surgeons missing cues given by patients, or a blinkered focus on the biomedical aspects of patient care [23, 24]. Furthermore, a 2015 public enquiry reported that more empathy is required in the NHS, since a lack of empathy led to failings in patient care in the Mid-Staffordshire Foundation Trust [25].

There is qualitative research into patient perceptions of surgeon empathy, but research with less of a focus on patient interactions and outcomes is needed [23, 26]. Indeed, the patient perspective is invaluable, but without knowledge of the practitioners’ thoughts and feelings, service improvement for the benefit of both staff and patients would not be possible.

### Study rationale

While there is literature demonstrating the decline in empathy, particularly in surgeons and its impact on patient care, there is little qualitative research on clinicians’ perceptions of this decline in empathy, or its perceived impact of this upon their practice. Furthermore, a large portion of the empathy research has taken place in the USA where there are important differences in medical practice compared to the UK, such as the funding system [26–31]. The qualitative research that has been done into empathy in surgery is also predominantly US-based and focuses on patient outcomes rather than practitioner perceptions or interprofessional relationships [22, 23, 26].

Additionally, most of the empathy research focusses on practitioners’ empathy towards patients, rather than for their colleagues. Little of the research on empathy in the UK focusses on surgical trainees, particularly in the form of interviews [32].

In the context of the NHS’ staffing crisis and high rates of attrition of doctors, this information could prove invaluable in practice as well as filling a crucial gap in the research [33]. Significant points of attrition of doctors include consultants retiring early and no longer opting to do extra sessions they used to in the past, and many medical students planning to work outside of the UK once qualified [34]. The COVID-19 pandemic put increased strain on already pressurised areas of the National Health Service, such as staffing shortages and increasing workload, as well as relationships between colleagues [35].

Qualitative insights into surgical culture are limited, as quantitative methods are often favoured in surgical research, and medical research more widely. The BMJ recently shared its stance on qualitative research as ‘low priority’, as these articles were less frequently accessed and cited, much to the dismay of many qualitative and mixed-methods researchers and others who had argued over many years of the important of understanding the value of systematic exploration of social, political and psychological issues in the training, delivery and organisation of health care [36, 37].

This research project aims to complement a growing body of quantitative information with qualitative insights that shed light on individual experiences and perceptions of empathy over the course of their career towards patients and between colleagues.

## Methods

### Research design and ethics

A qualitative study design was adopted, to explore the perceptions and experience of empathy for surgeons at different stages of training and to allow for inductive, interpretative analysis, which disputes the concept of a singular truth [38] but acknowledges the real-life impact of different subjective perspectives on social interaction and outcomes. Therefore, this research contributes to the wider field of interdisciplinary empathy research. Ethical approval was granted by the School of Liberal Arts and Natural Sciences ethics committee at the University of Birmingham in accordance with the University code of practice on research.

### Participants

To answer the research questions, surgical trainees were chosen because they have had experience working in surgical departments as a surgeon, but they are still in the midst of their training. In the UK, medical graduates complete 2 years of rotational foundation training, regardless of their desired specialty. The route to specialise in surgery is as follows: following completion of foundation training, they will enter core surgical training which consists of 2 years of rotational training through surgical specialties only. These are known as core surgical training year 1 (CT1) and core surgical training year 2 (CT2). Following this, they will enter specialty training (ST) which lasts approximately 6 years. After completion of specialty training, they receive a certificate of completion of training (CCT) and become a consultant surgeon [38].

The inclusion criteria were: participants were surgical trainees (CT1-2/ST1-ST8) in the UK NHS; they must have not yet achieved their CCT; they could be in an out-of-training job but must have commenced CT1 or ST1 and have plans to return to training, and they could be based anywhere in the UK.

#### Recruitment

The study flyer was disseminated on social media and via email to all surgical trainees on the West Midlands Deanery mailing list (which included a number of people who had since moved to other roles elsewhere in the country). The total number of surgical trainees in the West Midlands Deanery is approximately 500 [39]. Potential participants were asked to email the researcher for more information, before being asked for informed written or verbal consent to participate. Informed consent was obtained from all participants. No incentive was provided to participate, and recruitment stopped when data saturation occurred (*n* = 10).

#### Sampling

Initially a purposive approach was used, with the intention of recruiting a range of trainees of different ages, genders, and ethnicities to the study. However, to meet the recruitment target (reaching saturation), further snowball sampling was used (asking participants to recommend others, particularly those whose views might differ from their own). Finally, the study consisted of ten semi-structured interviews.

### Data collection

A topic guide was used and was used flexibly to allow for participants to raise unforeseen issues, as well as for the researcher to be able to adapt questioning based upon the participants’ comments. A pilot interview was carried out with a medical student, to test the answerability of the questions and whether they made sense to a clinical audience, but no substantial changes were required following this. The interviews lasted around 60 min and were digitally video recorded and transcribed by the researcher. Transcripts were anonymised prior to analysis.

### Data analysis

An inductive approach to analysis was used so that the themes were generated from the data, rather than being informed by prior theory. The Framework method for the analysis of qualitative data was used, which is a form of thematic analysis [40]. In line with inductive qualitative analysis principles, analysis took place alongside data collection, so that analysis could inform future interviews in an iterative process. All transcripts were coded by the first author, a medical student, and a lay person to facilitate and triangulate interpretations. Coded data were inputted into the Framework matrix and analytic memos were prepared for each emergent theme. These were discussed and debated with the second author, a social science researcher, prior to finalising themes.

## Results

Four main themes were identified in the data. The first was that being empathetic was a question of balance, the second was that the way an individual experiences empathy changes over their career (and is not always a decline), the third was that coping with COVID-19 has amplified aspects of team working, and the final theme was the view that generational change has resulted in societal changes in empathy perception. No new themes were emerging after ten interviews and Table 1 summarises the participants.

**Table 1:** Characteristics of study participants

ID	Gender	Age	Ethnicity	Grade	Specialty currently working in	Specialty desires to complete training in
1	Male	34	South Asian	ST6	General surgery (out of training- PhD)	Colorectal surgery
2	Male	27	South Asian	CT1	Trauma and Orthopaedic surgery	Trauma and Orthopaedic surgery
3	Male	32	South Asian	ST5/6	General surgery	General surgery/Upper Gastrointestinal
4	Female	33	White	ST5	General surgery (out of training- PhD)	General emergency and trauma surgery
5	Male	33	White	ST7	Paediatric surgery	Paediatric surgery
6	Female	43	Asian-Chinese	ST7	Maxillofacial surgery	Maxillofacial surgery
7	Female	29	White	CT1	General surgery	Unsure
8	Female	32	White	ST5	General surgery	Breast surgery
9	Male	28	South Asian	CT1	Trauma and Orthopaedic surgery	Plastic surgery
10	female	34	White	ST5	General surgery- breast	Breast/colorectal surgery

### Evolution of empathy in the individual: being empathic was a question of balance

#### Holism vs. efficiency

Participants all stated empathy was important in treating patients and often had a positive impact upon patient care. Many stated that part of empathising with their patients was understanding what was important to them and their families.

If you don’t understand what’s important to your patient, you will never be able to help them fully. - P8

Participants highlighted holism in the need to respect patient autonomy over paternalism and emphasized that getting to know their patients was crucial to identifying the best treatment option.

Actually, we’re here to treat you and, therefore, you should have a say in what we do to you. And that is absolutely correct- P2

The conflict between efficiency and comprehensive care was also mentioned by some participants. While they acknowledged the importance of empathy in treating patients, high volumes of patients and under-staffing can result in less time spent with patients and therefore the prioritisation of their clinical condition.

I saw like 46 patients in one twelve-hour shift. It’s just like fine just tell me, presenting complaint, like just running through the history, not really caring about what the patient says it’s more of just yes, no have you got these positive symptoms- P9

#### Objectivity vs. subjectivity

While participants all agreed that empathy was invaluable in treating patients, they suggested it was possible to empathise too much, and this could have negative implications for both the practitioner and patient. Participants also mentioned being criticised for showing emotions at work. This suggests a discomfort from colleagues towards demonstrating emotion in the surgical environment.

I have also received personal criticism for being too caring with my patients, for being too interested in what they have to say and for showing emotion when something… has affected me- P4

Other participants suggested that it was possible to focus too much on empathy and lose surgical quality. This indicates an implicit association between having a theoretical excess of empathy and deficiency in surgical skill, which is based on a binary system where empathy is compared to technical skill. This association may affect how people feel their colleagues perceive their surgical competence. This demonstrates an area where empathy between colleagues could be beneficial to making people feel less judged.

If you go too far the other way and make it all about empathy but then lose some aspects of surgical quality that’s also wrong- P3

Other participants saw being able to ‘switch off’ empathy as necessary to being effective in surgery, because being distracted by non-surgical factors can cloud one’s judgement. Participant 5 works in paediatric surgery, where the evidence base is limited, so the surgeons’ clinical decisions often take precedence [41]. In these situations, the participant stated it is important not to let emotions impact treatment decisions.

you have to have the ability to be focused on surgery to be able to be objective, to be able to make good decisions… without feeling bad about yourself for making someone intentionally worse with the aim of making them better-P5

Therefore, there are three primary reasons participants cited for needing to remain as objective as possible and not empathising ‘too much’: intrinsic depletion of emotional resources, contamination of the patient’s plan with one’s own emotions and fear of criticism by colleagues. These are both intrinsic and extrinsic reasons.

#### The Team vs. the individual

Participants mentioned trying to be empathetic towards patients can result in negative impacts upon the team, demonstrating the delicate balance between looking after patients, and being considerate towards colleagues.

if I give too much time to one patient then the whole department could lose their lunch break- P6

They highlighted the competitive nature of surgery and how this contrasts with the need to work in a team, which can affect working relationships. Participants stated it is sometimes difficult to ask for help from colleagues as they would be talked about or perceived as less competent; this dynamic undermines team working.

we need to try and remove some of the competitive nature of specialty training… Because you know you can’t show anybody else if you were struggling with a particular aspect of your training it’s seen as a weakness- P7

### Empathy changes over career

Participants spoke of how the way they empathise with patients has changed over their years of practice. Here, this qualitative evidence paints a more complex picture than the previous quantitative data, which simply shows a decline in empathy over time. Participants cited two reasons for this change: experience with patients at work and experience in their personal life. For example, some participants became parents over the course of their training, and that has changed the way they understand the way paediatric patients’ parents feel. Other participants, who had experiences with illness in their personal lives described feeling more emotionally impacted when they see patients who remind them of this situation.

I see myself as able to empathize with that parent because I’m also a parent- P3

However, participants associated increased experience of the job with becoming desensitised. The desensitisation was not always associated with a decrease in empathy, though it was for some participants. Participants did not think the clinical care they provided was made worse by this change, and in fact felt it was beneficial as it would be unsustainable to be deeply emotionally affected by every patient.

I get less personally upset by seeing bad things happen to patients but I’m still able to empathize with them and their family.- P3

Here we can see it is not simply a question of empathy declining, but of it evolving over the course of an individual’s career.

### Evolution of empathy in response to extreme events: coping with COVID-19 has amplified the positive and negative

Participants also discussed how the COVID-19 pandemic specifically had an impact upon their wellbeing at work and ability to empathise with patients. Most participants referred to burnout and how the pandemic had worsened this. They referred to the impact of not having respite from continuous work throughout the pandemic, but also the psychological burden of being seemingly undervalued. This was compounded by the trauma of what participants saw during the pandemic.

…burnt out as a consequence of untapped care being poured out, with no reward no relief- P5

However, one participant identified that at her hospital, teamwork improved during the pandemic as everyone came together with a shared understanding of what their colleagues were going through.

The teamwork was the best it’s ever been it was very, especially at the start of the pandemic, It was almost like people were being very supportive to one another being very kind to one another- P7

Other participants felt COVID has a negative impact upon teamwork as staff absence due to COVID infection put a strain on the rest of the team.

More often recently, you know we’ve had absences in the team it’s very easy for in a poor team culture to start blaming those absences- P2

Furthermore, many trainees experienced vastly reduced opportunities to gain operating experience in theatre due to redeployment, the cancelling of elective surgeries, and the reduced number of surgeries that can be carried out in a day because of COVID cleaning procedures. Participants stated this had a negative impact upon their training, with some participants stating it increased competitiveness between colleagues further.

what COVID has done is that because there’s been a year and a half, lack of elective and also emergency theatres, trainees have become a lot more cutthroat-P10

### Generational evolution of empathy

Participants stated there was a generational change in the attitudes of doctors, particularly seniors towards patients and colleagues. They observed a shift from more paternalistic, authoritarian views towards a more flexible, tolerant one.

at the beginning of my training when I would see seniors or juniors, for example, not necessarily consenting patients thoroughly before intimate examinations- P3

Participants noted that newer consultants who are qualifying are more empathetic than their predecessors.

there is a definite change in terms of younger consultants who are more accepting and empathetic that seems to be a better trend.-P4

Some participants attributed this change to an alteration in the types of people favoured in surgical selection. While previously interpersonal skills were not necessarily valued in surgical selection, they are now being weighted more heavily.

you’re going into surgery, because you think that you can be a bully you know, then you’re not going to get very far anymore.- P7

We are now looking for consultants who will mentor, educate, and support their juniors.- P8

Some participants linked this change in desirable attributes to a general societal change whereby people are becoming more empathetic towards one another. This shows that as well as a change in empathy over an individual’s career and life, societal change can impact the way people empathise on a generational level.

The world has become more sensitive to people’s needs and the importance of, for example, mental health- P4

While participants associated more progressive attitudes in medicine and surgery with a general societal change, others were not convinced, and saw changes within surgery as more superficial and performative.

I hear sexist comments a lot when women aren’t around… It’s like oh thank God she’s gone, like you know at least you can hold the camera steady- P9

Participants did feel sexist and racist attitudes prevailed, but in a more subtle way than they might have been in the past (although the above quote may not be considered so). Both male and female participants highlighted this, but male participants seemed to be privy to sexist comments as participants suggested the perpetrators are aware this behaviour is inappropriate, and therefore try to conceal it.

Furthermore, other participants commented on racist behaviour they had witnessed or been the target of, further suggesting that discrimination in medicine and surgery is far from gone.

I’m Chinese and there was a lot of anti-Chinese sentiment during COVID so there was a lot of abuse, aggression and I had colleagues that were also a little bit abusive as well- P6

While the participant quoted above describes explicit racism, participants also described witnessing racism in the form of microaggressions, and conflicts where an individual’s race may have played a part in how they were treated. The below quotation reflects a reluctance to name racism as a factor in workplace conflicts alongside an awareness that it could be.

I don’t think race was involved, so I don’t want to delve into that, I don’t think it was involved, but you never know- P1

## Discussion

The very generation of qualitative research on the experience of surgical trainees in the UK is novel, since they are a previously unresearched study population using qualitative methodology in the UK. This research contributes experiences of a change in empathy towards patients, rather than a specific decline among UK surgeons, as well as their experiences of interprofessional empathy. Further contributions include the impact of the COVID 19 pandemic on relationships within the medical team and on training opportunities.

### Empathy towards patients evolves over time and experience

The most commonly used tool for measuring empathy is the Jefferson Scale of Empathy (JSE) questionnaire. As mentioned above in *Empathy Decline in Surgeons*, the JSE shows a decline in empathy in doctors over their careers [13–17]. The JSE has been validated in multiple ways [28, 42]. While JSE responses show strong evidence for a decline in empathy, it is unclear whether the questionnaire captures the distinction between desensitisation and an actual decline in empathy. Additionally, the JSE does not provide explanations for *why* doctors and medical students feel less empathetic towards patients, which limits the data’s utility.

Empathy towards patients was not seen as lacking overall by participants, and this could be because participants experienced a change in empathy towards patients rather than a decline in it, as quantitative data suggests. While participants referred to a desensitisation that occurs over the course of their careers, many of them explicitly said that they feel *more* empathy towards patients now than they did earlier in their careers, because of more life experience and experience with patients, as well as feeling they have a more significant role in the patient’s care. Furthermore, participants described the need to disconnect somewhat while undertaking surgery, because they are ultimately causing physical trauma to the patient with risks involved. This is supported in Han and Pappas’s paper which describes how surgeons need a ‘self-defence’ mechanism in the face of the traumatic nature of their healing [21]. They also discuss a “distinctly surgical empathy”, which could be a downregulation of the affective response in order to diminish “counterproductive” emotions such as fear [43]. Han and Pappas suggest that rather than surgeons being ‘less empathetic’, they need a degree of suppression in order to minimise the emotional impact of the trauma they inflict as part of their healing to avoid compassion fatigue, burnout, and the consequences of these [21, 44]. This links to what participants said about remaining objective and how the concept of empathising ‘too much’ could have negative repercussions.

The fact that this study’s results do not support JSE responses is not necessarily a limitation of the research, as it provides an insight into the complexity of the topic and captures different truths that were not previously recorded in research, namely the distinction between desensitisation and decline in empathy and this being a possible reason for incongruence between results of this study and JSE responses [45].

### Interprofessional relationships also require empathy

While empathy towards patients was not seen as lacking by most participants, empathy towards colleagues was highlighted as an area that could be improved.

Participants felt that competition in their workplace resulted in a negative impact on their training, as they did not feel able to ask for help from colleagues if they needed it for fear of being seen as weak or being gossiped about. When asked why they thought surgical training is so competitive, participants attributed it to being a male-dominated environment or the scope for private practice. This is compelling as these are factors that pertain to the characteristics of people that enter surgical training, rather than, for example, organisational structures and funding issues that result in bottlenecking, and therefore competition, despite there being a shortage of doctors. The answer may be that the characteristics of people in an organisation influence its structure, which in turn influences people’s behaviours [46].

Participants felt competition had a negative impact on their training. This raises the question of whether competitiveness is conducive to teamwork and collaborative learning. The traditional belief that competition is a prerequisite for surgical training is being challenged.

When discussing ways to improve empathy between colleagues, participants emphasised getting to know colleagues through having consistent teams. They suggested this could also be a way to lighten the competitive atmosphere. Having consistent teams has been suggested in previous research as a way to support junior doctors through trying experiences, such as the COVID-19 pandemic [35].

### The COVID-19 pandemic resulted in an erosion of empathy

Participants highlighted the impact of COVID-19 on their training, shedding light on a new challenge post-pandemic. While some described the empathy erosion they experienced as a result of the volume of death they saw during the pandemic, they also spoke of the lack of respite from work, as many trusts cancelled staff’s annual and study leave. Participants related these issues to feelings of burnout, which is characterised by feelings of “mental exhaustion, depersonalization, and a decreased sense of personal accomplishment” and although fairly common, it should not be underestimated, as burnout is associated with adverse outcomes such as depression, substance abuse, suicide, and attrition [47]. While burnout was an issue pre-pandemic, its incidence has drastically increased since the pandemic, and this is demonstrated in literature investigating the impact of COVID-19 on frontline workers’ mental health [48–51]. These studies focus on the short-term impact of the pandemic, particularly burnout. This study, however, presents novel findings on surgical trainees’ perceptions of the longer-term impacts of COVID upon their training, such as reduced theatre experience, and the ongoing staff shortages due to self-isolation, specific to the UK. Participants’ responses suggest a need for further specific research into the impact this will have on both trainee wellbeing, and training outcomes. Training requirements have not changed in response to the impact of COVID-19, meaning many trainees will have to extend their training in order to meet operating targets. While trainee organisations have published education guidance, they do not necessarily influence policy. Furthermore, as participants mentioned, if senior surgeons do not look at this guidance, they have no awareness of what their trainees need and cannot support their learning [52, 53].

### Strengths and limitations

The qualitative data this study provided enabled discovery of previously undocumented findings on the nature of empathy change over an individual’s career, which complements quantitative data.

There was some participation bias present in recruitment of participants for the study because those who took part were interested in empathy and self-reflection. Therefore, this could have impacted upon the results whereby the majority of the participants did not perceive a straightforward decline in empathy, but a more nuanced picture.

There may be more variation in perspectives and experiences than we were able to capture in this particular dataset, particularly if this study was repeated in other regions or nations, or with surgeons at later stages of their careers. Therefore, transferability may be a limitation of this study.

Social desirability bias is a “tendency to present reality to align with what is perceived to be socially acceptable” [54]. The interviews were face-to-face, so as participants could see the researcher’s reactions to what they were saying, they may have been less likely to provide her with answers that could elicit negative judgement [55].

The limitations of this study highlight areas for possible future studies. A potential study could use anonymised surveys with free-text boxes, but these come with their own limitations, including bias and a scarcity of detailed data [56]. Alternatively, a mixed-methods project combining JSE responses and interviews could be carried out, but this is not feasible for a student-led project as the JSE is not available freely online; it must be purchased to be accessed [57]. Although there is much literature validating it, this limits criticism of it since it is not possible to access the official questionnaire without purchasing it.

## Conclusion

To conclude, this research provides an insight into surgical trainees’ perceptions around empathy in their practice. Of particular importance is the distinction participants made between a desensitisation and actual decline in empathy, with participants stating they experienced more of the former. The data generated from this study is novel, as surgical trainees are a previously little-researched study population, particularly in the UK. They are important because they provide a detailed insight into surgical training in the UK, and particularly the longer-term impacts of previous structural changes as well as the effects of the COVID-19 pandemic on training.

## Data Availability

The datasets used and/or analysed during the current study are available from the corresponding author on reasonable request.

## Abbreviations

CT: core trainee
ST: specialty training
